# Comparison of the Dentin Bond Strength of Two Self-Etch Adhesives After Prolonged Air-Drying and Additional Light-Curing

**Published:** 2017-09

**Authors:** Pouran Samimi, Masoud Ghodrati, Farinaz Shirban, Maryam Khoroushi

**Affiliations:** 1 Associate Professor, Dental Materials Research Center, Department of Operative Dentistry, School of Dentistry, Isfahan University of Medical Sciences, Isfahan, Iran; 2 Assistant Professor, Department of Operative Dentistry, School of Dentistry, Urmia University of Medical Sciences, Urmia, Iran; 3 Assistant Professor, Dental Research Center, Department of Orthodontics, School of Dentistry, Isfahan University of Medical Sciences, Isfahan, Iran; 4 Professor, Dental Materials Research Center, Department of Operative Dentistry, School of Dentistry, Isfahan University of Medical Sciences, Isfahan, Iran

**Keywords:** Shear Strength, Dental Adhesives, Time, Light-Curing of Dental Adhesives

## Abstract

**Objectives:**

It has been reported that the water, solvents, or the primer incorporated into adhesive resins decrease the polymerization, compromise the mechanical properties, reduce the bond strength, and lead to a poor bonding performance of self-etch adhesives. This article evaluated the effect of air-drying and light-curing duration of self-etch adhesives on the micro-shear bond strength between composite resin and dentin.

**Materials and Methods:**

A total of 120 extracted sound human third molars were randomly divided into twelve groups (n=10). The occlusal dentin in each tooth was exposed. Clearfil SE Bond (CSEB) and Clearfil S3 Bond (CS3B) were used according to the manufacturer’s instructions, followed by air-drying for 3 and 10 seconds in different groups. The adhesives were light-cured for 10, 20 and 40 seconds in different subgroups. Next, the composite resin (Clearfil AP-X) was placed on the dentin surface and was polymerized for 40 seconds. The micro-shear bond strength values were determined using a universal testing machine, and the results were statistically analyzed by three-way ANOVA and Tukey’s post-hoc test (α=0.05).

**Results:**

CSEB exhibited a significantly higher dentin bond strength than CS3B. Increasing the curing time of CSEB resulted in an increase in the bond strength, whereas an increase in the air-drying time did not affect the bond strength of the two adhesives.

**Conclusions:**

Within the limitations of this study, an increase in the curing time improved the bond strength of CSEB, whereas the air-drying time did not affect the bond strength of the evaluated adhesives.

## INTRODUCTION

One-step self-etch adhesive systems exhibit low technique sensitivity and consistent performance due to their simplified application procedures; however, there are controversies over their performance [1]. Adhesives contain solvents such as water, ethanol, or acetone to dissolve the monomers, preserve the expanded state of the collagen network and allow the monomers to fill the gaps in and around the collagen fibrils. The chemical polymerization of these monomers, activated by the curing light, yields a polymer-collagen bio-composite, generally referred to as the “hybrid layer”. However, irrespective of the applied system or material, the hybrid layer is not perfect [2,3]. The presence of water, which is necessary for keeping the collagen network open for resin penetration, and the limited degree of conversion (polymerization rate) are the most important obstacles that usually prevent the formation of a uniform dentin bond [4]. The bond strength of self-etch adhesive systems is affected by variables such as the enamel surface treatment method, thickness of the smear layer, bur grit size, the moisture of the adhesive surface, drying duration after applying the adhesives, number of coatings, and light-curing duration [5–8]. Excessive moisture may result in phase separation between hydrophobic and hydrophilic monomers, giving rise to a non-uniform resin infiltration and formation of bubbles and voids at the bonding interface [4]. It has been proposed that the water, solvents, or the primer in the chemical structure of adhesive resins compromise the mechanical properties and result in a poor bonding performance; therefore, removal of these components appears to have beneficial effects [9]. Defining a certain criterion for sufficient air-drying appears to be difficult and practitioners have to compromise to achieve the best results, especially with all-in-one systems [10]. It seems that elimination of water and solvents from the adhesive layer by prolonged air-drying, and also improving the polymerization of resin monomers by increasing the irradiation time can increase the bond strength and improve the mechanical properties of the adhesive layer [9, 11].

In different studies, extended air-drying of the adhesive has been suggested to decrease residual water in the adhesive layer. It has also been suggested to increase the light-curing duration to enhance the degree of polymerization of the adhesive layer [6, 11–13]. In another study, it was concluded that delayed composite resin curing diminishes the dentin bond strength of the single-step self-etch adhesive [14]. Cadenaro et al [11] showed that OptiBond FL, Clearfil Protect Bond, and Xeno III adhesives may become less permeable by using longer curing times than those recommended by the manufacturers. Ikeda et al [13] revealed that a significant decrease in the ultimate tensile strength of OptiBond FL adhesive resin, when mixed with the primer, can be attributed to the incomplete evaporation of primer components. It is difficult to achieve total evaporation of solvents even by complete air drying.

All the contemporary ‘simplified adhesives’ used in self-etch adhesive systems contain mixtures of hydrophilic monomethacrylates and additional hydrophobic dimethacrylates to provide sufficient cross-linking and create strong bonding agents. Through careful formulation, the manufacturers incorporate sufficient solvents to produce a single-phase solution. The ultimate aim is to make the hydrophilic and hydrophobic comonomers copolymerize to create uniformly cross-linked copolymerized chains. However, when the bonded resins are stained by ammoniacal silver nitrate and are examined under a transmission electron microscope, the resin films do not appear to be homogeneous; rather, they exhibit water-filled voids and channels referred to as “water trees” [15]. Insufficiently-cured adhesives are more permeable than optimally-cured adhesives [16,17]. Under-curing might be attributed to inadequate irradiation, dilution by excessive solvent, or a low rate of solvent evaporation [18]. The current study aimed to evaluate the micro-shear bond strength of one-step and two-step adhesives to dentin after different air-drying and light-curing durations. Scanning electron microscopy (SEM) was performed to observe the resin-dentin interface.

## MATERIALS AND METHODS

A total of 120 freshly extracted sound human third molars were evaluated in the present study. The teeth had no caries, restorations, or cracks, and were stored in 0.02% thymol solution immediately after extraction. The teeth were randomly divided into 12 groups (n=10; Table 1). The enamel on the occlusal surfaces of the teeth was removed with the use of diamond fissure burs (D+Z, Diamant GmbH, Germany) under air and water spray. Next, the dentin surfaces were polished by Sof-Lex discs (3M ESPE Dental Products, St. Paul, MN, USA). In Clearfil SE Bond (CSEB, Kuraray, Osaka, Japan) groups (groups 1 to 6), the relevant primer (Kuraray, Osaka, Japan) was applied on the dentin surfaces for 20 seconds according to the manufacturer’s instructions, followed by air-drying for 3 seconds (groups 1 to 3) and 10 seconds (groups 4 to 6) in each group at a distance of 5cm from the surface of the sample using an air syringe with oil-free compressed air at a pressure of 4 kg/cm^2^. Finally, the adhesive (Kuraray, Osaka, Japan) was applied according to the manufacturer’s instructions and was light-cured for 10, 20 and 40 seconds using the continuous mode of the Bluephase (Ivoclar Vivadent AG, FL-94941: Lichtenstein) light-curing device with the light intensity of 580mW/cm^2^. In Clearfil S^3^ Bond (CS3B, Kuraray, Osaka, Japan) groups (groups 7 to 12), the bonding agent (Kuraray, Osaka, Japan) was applied on the prepared dentin surfaces for 20 seconds with the use of a spongy micro brush, based on the manufacturer’s instructions, followed by air-drying and light-curing procedures in a manner similar to those in CSEB groups. After the completion of the bonding procedures, a Tygon® tubing (Norton Performance Plastic, Saint-Gobain, Akron, USA) with 1mm height and 0.7mm diameter, filled with composite resin (Clearfil AP-X, Kuraray, Osaka, Japan, A3 shade), was cured on the adhesive layer for 40 seconds with the use of the Bluephase light-curing unit. Afterwards, the samples were stored in water at 37°C for 24 hours. Each dentin segment containing the cylindrical composite was placed in a universal testing machine (Dartec, HC10, Stourbridge, UK) for micro-shear bond strength testing. A shearing force was applied to the samples at a crosshead speed of 0.5 mm/minute with a blade measuring 0.4mm in thickness until the cylindrical composite resin fractured. One sample of each group was prepared for evaluation under a scanning electron microscope (SEM, Philips, XL30, The Netherlands) [19]. Data were analyzed with three-way ANOVA followed by Tukey’s post-hoc test to determine the significance of the differences.

**Table 1.** Means, standard deviations (SD) and 95% confidence intervals of the micro-shear bond strengths (MPa)

**Adhesive**	**Group number**	**Air-drying time (s)**	**Light-curing time (s)**	**Mean (MPa)**	**SD**	**95% Confidence Interval**									

						**Lower Bound**	**Upper Bound**	**Minimum**	**Maximum**
**Clearfil SE Bond**	1	3	10	10.13	8.17	4.29	15.98	2.60	26.00
	2		20	17.67	5.17	13.97	21.37	7.80	26.00
	3		40	22.35	10.12	15.11	29.59	5.20	39.00

	4	10	10	16.89	9.43	10.15	23.64	2.60	39.00
	5		20	20.79	9.80	13.78	27.81	5.20	36.40
	6		40	22.87	9.23	16.27	29.48	10.40	36.40

**Clearfil S^3^ Bond**	7	3	10	9.35	5.90	5.13	13.58	2.60	20.80
	8		20	11.43	5.90	7.21	15.66	2.60	23.40
	9		40	15.59	10.10	8.36	22.82	5.20	33.80

	10	10	10	9.09	5.51	5.15	13.04	2.60	20.80
	11		20	10.65	7.08	5.58	15.72	2.60	23.40
	12		40	11.69	5.64	7.65	15.74	2.60	18.20

## RESULTS

The means, standard deviations and 95% confidence intervals of the micro-shear bond strength values in each group are presented in Table 1. CSEB exhibited a significantly higher dentin bond strength than CS3B (P<0.001). Three-way ANOVA showed that the adhesive type (P<0.001) and light-curing time (P=0.001) had significant effects on the shear bond strength, whereas the air-drying time had no significant effect on the shear bond strength (P=0.53). The interaction effects between the variables, adhesive type versus air-drying time (P=0.08), adhesive type versus light-curing time (P=0.37), air-drying time versus light-curing time (P=0.37), adhesive type and air-drying time versus light-curing time (P=0.89), were not significant. Three-way ANOVA followed by Tukey’s post-hoc test showed that the difference was significant only between 10- and 40-second light-curing times (P=0.001).

**Fig. 1:**
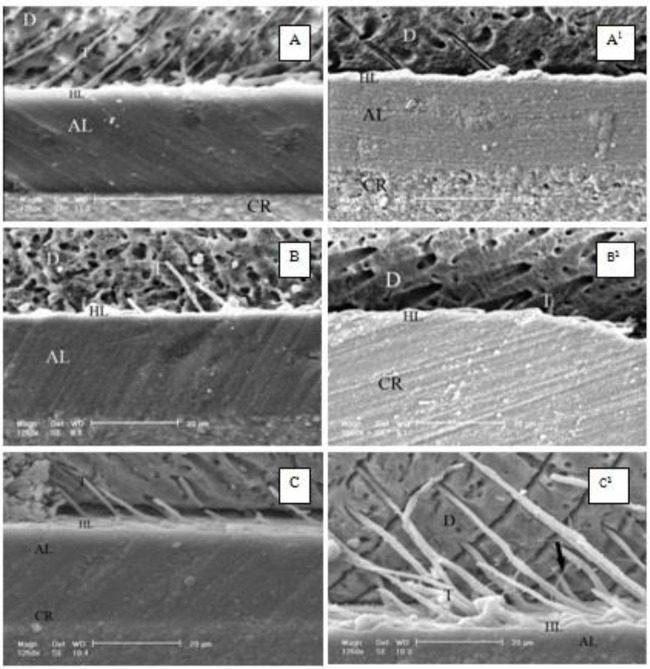
The dentin-Clearfil SE Bond interface. (A) air-drying time: 3s, light-curing time: 10s. (A1) air-drying time: 10s, light-curing time: 10s. (B) air-drying time: 3s, light-curing time: 20s. (B1) air-drying time: 10s, light-curing time: 20s. (C) air-drying time: 3s, light-curing time: 40s. (C1) air-drying time: 10s, light-curing time: 40s. D=Dentin, HL=Hybrid Layer, T=Resin Tag, AL=Adhesive Layer, CR=Composite Resin

**Fig. 2:**
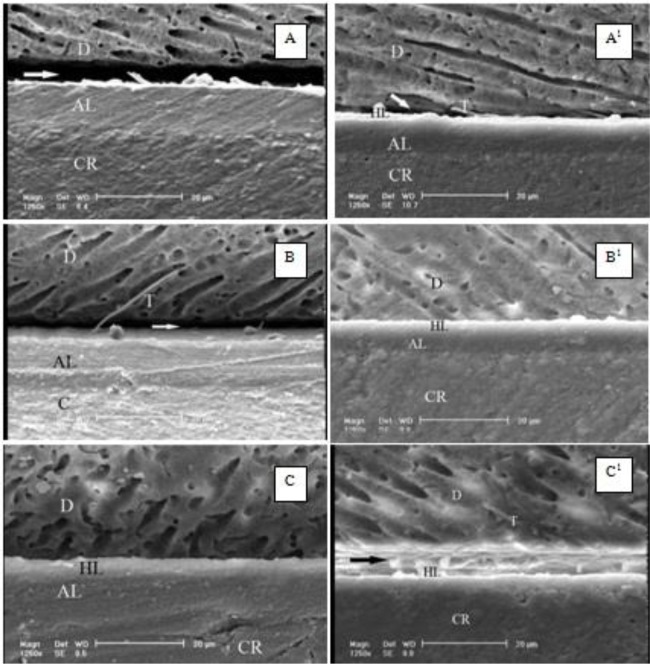
The dentin-Clearfil S3 Bond interface. (A) air-drying time: 3s, light-curing time: 10s. (A1) air-drying time: 10s, light-curing time: 10s. (B) air-drying time: 3s, light-curing time: 20s. (B1) air-drying time: 10s, light-curing time: 20s. (C) air-drying time: 3s, light-curing time: 40s. (C1) air-drying time: 10s, light-curing time: 40s. D=Dentin, HL=Hybrid Layer, T=Resin Tag, AL=Adhesive Layer, CR=Composite Resin

The P-values obtained by pairwise comparisons using Tukey’s post-hoc test are shown in Table 2. By increasing the light-curing time from 10 seconds to 40 seconds, the dentin micro-shear bond strength increased in both adhesive groups, which was significant only with regard to CSEB (P=0.036 between groups 1 and 3). The SEM images (Fig. 1 and 2) showed that the adhesive layer of CS3B was thinner than that of CSEB. Also, a decrease in the thickness of the adhesive layer in CS3B was noticed with an increase in the air-drying time.

**Table 2.** Significant P-values between the groups (Tukey's HSD test)

**Group number**														

**Adhesive**	**Group number**	**Air drying time (s)**	**Light curin time (s)**	**1**	**2**	**3**	**4**	**5**	**6**	**7**	**8**	**9**	**10**	**11**
**Clear fil SE-Bond**	1	3	10	-	0.602	0.036	0.750	0.119	0.023	1.000	1.000	0.925	1.000	1.000
	2	3	20	0.602	-	0.974	1.000	0.999	0.945	0.447	0.833	1.000	0.399	0.703
	3	3	40	0.036	0.974	-	0.925	1.000	1.000	0.018	0.099	0.750	0.014	0.055

	4	10	10	0.750	1.000	0.925	-	0.994	0.869	0.602	0.925	1.000	0.550	0.833
	5	10	20	0.119	0.999	1.000	0.994	-	1.000	0.067	0.268	0.945	0.055	0.168
	6	10	40	0.023	0.945	1.000	0.869	1.000	-	0.011	0.067	0.653	0.009	0.036

**Clear fil S^3^-Bond**	7	3	10	1.000	0.447	0.018	0.602	0.067	0.011	-	1.000	0.833	1.000	1.000
	8	3	20	1.000	0.833	0.099	0.925	0.268	0.067	1.000	-	0.990	1.000	1.000
	9	3	40	0.925	1.000	0.750	1.000	0.945	0.653	0.833	0.990	-	0.794	0.962

	10	10	10	1.000	0.399	0.014	0.550	0.055	0.009	1.000	1.000	0.794	-	1.000
	11	10	20	1.000	0.703	0.055	0.833	0.168	0.036	1.000	1.000	0.962	1.000	-
	12	10	40	1.000	.869	.119	.945	.309	.082	1.000	1.000	0.994	1.000	1.000

## DISCUSSION

Based on the results of the micro-shear bond strength tests, CSEB formed a more effective bond to dentin than CS3B adhesive. CS3B has a higher pH than CSEB (2.7 versus 1.9); as a result, it is expected to dissolve and remove less dentinal mineral content. On the other hand, CSEB is a two-step self-etching bonding agent with a hydrophilic aqueous primer that should be air-dried for elimination of water and solvent contents before adhesive application. CSEB contains a hydrophobic resin with no water or solvent in its chemical structure; however, in CS^3^B, as for the one-step self-etching systems, a significant amount of water and solvent is included in the adhesive container, which is expected to be removed by air-drying after the application of the adhesive. Considering the harmful effects of the residual water and solvents on the bonding performance [6, 10, 20], many studies have demonstrated that water trees, voids, water droplets, and phase separation occur at the adhesive interface of one-bottle and all-in-one adhesive systems [15, 21,22].

An increase in the light-curing duration rendered increased bond strength, which was statistically significant in CSEB groups. Previous studies on this subject have shown that overall, an increase in the curing time of self-etch adhesives results in an increase in the polymerization degree, decreased permeability, and improved mechanical properties of the adhesive layer, all of which can improve the dentin-adhesive bond strength [8, 11, 18]. In addition, a number of studies have shown a decrease in the thickness of the oxygen-inhibited layer with prolonged curing time [23,24]. The high level of energy received from light-curing might result in higher polymerization of the chain and progression of the polymerization toward the oxygen-inhibited layer, which leads to decreased thickness of this layer. In fact, with prolonged curing time, the degree of conversion of resin monomers increases, while the thickness of the oxygen-inhibited layer decreases, leading to improved mechanical properties and increased adhesive-dentin bond strength [23,24].

Extended drying of the adhesive layer has been suggested in several studies in order to decrease the water and solvent contents in this layer [6, 12,13]. In the present study, increasing the air-drying time in CS^3^B groups decreased the bond strength, though this decrease was not significant. The increased bond strength of CSEB groups after longer air-drying durations may be due to the greater elimination of water and solvents from the resin-dentin interface, although the increase of bond strength was not significant. Some studies have shown the correlation between the adhesive layer’s thickness and the efficacy of bonding [25]. It seems that extended air-drying of CS^3^B might decrease the thickness of the bonding layer, which might be a reason for the reduced bond strength of the adhesive with an increase in the air-drying time from 3 seconds to 10 seconds. Comparison of the SEM images shows that the adhesive layer of CS^3^B is thinner than that of CSEB (Fig. 1 and 2). Also, a decrease in the adhesive layer thickness in CS^3^B was observed with prolonging the air-drying time from 3 seconds to 10 seconds (Fig. 2). In addition, the gaps detected on the SEM images at the resin-dentin interface in CS^3^B groups might explain the weaker bond of this adhesive compared to CSEB (Fig. 2). On the other hand, the SEM images of CSEB revealed more resin tags compared to CS^3^B (Fig. 1 and 2). However, a large number of resin tags cannot be considered a reason for a higher bonding quality. In addition, a further explanation for the weaker bond of CS^3^B adhesive compared to CSEB might be the saturation of the adhesive layer with oxygen, which inhibits the polymerization of resin monomers. Oxygen is a strong inhibitor of methyl methacrylate polymerization. Oxygen reacts with the carbon-based polymerizing free radicals in a diffusion-controlled manner to form peroxy radicals, which exhibit low reactivity toward double bonds and significantly delay the polymerization reaction [26]. It has been reported that an adequate thickness of the oxygen inhibition layer is necessary for bonding composite resins to adhesives [22]. Furthermore, a non-polymerized adhesive surface was reported to have no effect on the bonding to the overlying composite resin [27,28]. In addition, a number of studies have shown that a decrease in solvents in the adhesive layer results in a significant increase in the degree of polymerization and the thickness of the oxygen-inhibited layer, which might be attributed to the increased viscosity of the adhesive layer due to the removal of solvents, resulting in a limited penetration of oxygen into the viscous adhesive layer [23, 29].

## CONCLUSION

Within the limitations of the present study, an increase in the curing time improved the bond strength of Clearfil SE Bond to dentin; however, the air-drying duration did not affect the dentin bond strength of the evaluated adhesives.
